# Assessment of function, histopathological changes, and oxidative stress in liver tissue due to ionizing and non-ionizing radiations

**DOI:** 10.22088/cjim.11.3.315

**Published:** 2020-05

**Authors:** Sajad Borzoueisileh, Ali Shabestani Monfared, Hossein Ghorbani, S. M. J. Mortazavi, Ebrahim Zabihi, Mehdi Pouramir, Amir Hossein Doustimotlagh, Mohsen Shafiee, Fatemeh Niksirat

**Affiliations:** 1 Cellular and Molecular Biology Research Center, Health Research Institute, Babol University of Medical Sciences, Babol, Iran; 2 Student Research Committee, Babol University of Medical Sciences, Babol, Iran; 3 Cancer Research Center, Health Research Institute, Babol University of Medical Sciences, Babol Iran; 4 Pathology Department, Babol University of Medical Sciences, Babol, Iran; 5 Department of Medical Physics, School of Medicine, Shiraz University of Medical Sciences, Shiraz, Iran; 6 Medicinal Plants Research Center, Yasuj University of Medical Sciences, Yasuj, Iran; 7 Cellular and Molecular Research Center, Yasuj University of Medical Sciences, Yasuj, Iran; 8 Department of Medical Physics Radiobiology and Radiation Protection, School of Medicine, Babol University of Medical Sciences, Babol, Iran

**Keywords:** EMF, Ionizing radiation, Liver, liver function tests, Oxidative stress

## Abstract

**Background:**

Compared to past decades, humans are exposed to rapidly increasing levels of radiofrequency electromagnetic radiations (RF-EMF). Despite numerous studies, the biological effects of human exposure to different levels of RF-EMF are not fully understood yet. This study aimed to evaluate the bioeffects of exposure to "900/1800 MHz" and “2.4 GHz" RF-EMFs, and x-rays alone as well as their potential interactions, i.e. inducing simple additive, adaptive, or synergistic effects.

**Methods:**

120 Wistar rats were randomly divided into ten groups of 12 each. The rats were exposed to RF-EMF, 10 cGy, and 8 Gy x-rays, a combination of these exposures, or only sham-exposed. The levels of liver enzymes were determined in serum samples by an auto-analyzer. Moreover, the histopathological changes, and the levels of malondialdehyde (MDA), nitric oxide, ferric reducing antioxidant power, total thiols, and protein carbonyl (PCO) were measured.

**Results:**

Among the markers of liver function, gamma-glutamyltransferase was not associated with irradiation but, aspartate transaminase, alanine transaminase, and alkaline phosphatase showed some levels of association. MDA and PCO levels after 8 Gy irradiation increased, but pre-exposure to RF-EMF could modulate their changes. At the cellular level, the frequency of lobular inflammation was associated with the type of intervention.

**Conclusion:**

The exposure to both ionizing and non-ionizing radiations could alter some liver function tests. A short term pre-exposure to RF-EMF before exposure to an 8 Gy challenging dose of x-rays caused the alterations in oxidative stress markers and liver function tests, which indicate that oxidative stress is possibly involved in the adaptive response.

The liver is the largest organ in the body and located in the right upper quadrant of the abdomen (1). This endocrine gland has several functions, including involvement in the metabolism process of lipids and bile production (2). Numerous environmental conditions, such as ionizing and non-ionizing radiations, could interfere with liver functions and disturb the tissue structure (3). Ionizing radiations are electromagnetic waves or particles which could produce ions that may lead to chemical interactions in the target tissue (4). These radiations are widely employed in the medical and industry. Non-ionizing radiations are electromagnetic waves, which are not able to ionize the target but could trigger a change in the cells by thermal and non-thermal effects (5). The frequencies between 300 MHz and 300 GHz of non-ionizing radiations, including mobile phones and Wi-Fi routers and industry, are widely used in daily work (6). 

The uses of both types of ionizing and non-ionizing radiations are inevitable, but numerous reports are dealing with their harmful effects on the animal and human body. Ionizing radiation could trigger a chain of reactions in the liver, which begins with ionization, and DNA breaks, continue with molecular and cellular changes, finally lead to liver diseases and clinical manifestations at organ level and body level. A review in the literature showed that ionizing radiation could lead to significant changes in the liver metabolomes (7), cancer (3), mild steatosis, necro-inflammation and fibrosis, and many other pathological properties (8). The changes at the molecular level could be, but not limited to, ion production and oxidative stress, and cytogenetic changes. However, there are compensatory mechanisms in the body, such as activation of repair mechanisms and anti-oxidative abilities, which could modulate these adverse effects (3). Some of these mechanisms are common in different stressors. For example, radiofrequency electromagnetic fields (RF-EMFs) such as mobile phones and Wi-Fi radiations and also, x-ray, could induce oxidative stress and alter genes expression (9). Nevertheless, the lack of information about the exact mechanisms of RF-EMF effects is evident. Furthermore, the simple additive, adaptive, or synergistic effects of RF-EMFs with ionizing radiations are not identified in detail.

The adaptation response (AR) could occur when the body modulates the harmful effect of challenging doses of ionizing radiation. This adaptation to ionizing radiation could be achieved by an adaptive dose of ionizing radiation or RF-EMF (10). There is a dose window, which could induce adaptation, but there are many doubts about the mechanisms of this phenomenon. The interphone study group has suggested that mobile phones did not have the risk of glioma or meningioma, and they suggested the further study of long-term heavy use of mobile phones (11), but there is some evidence implying a decrease in brain diseases due to AR (12). Contrariwise, in 2011, WHO declared that EMF is classified as a possibly carcinogenic agent based on brain cancer evidence in the long term of mobile phone use (9). The lack of evidence about the effects of RF-EMFs on the liver and the existence of AR is apparent.

The function of the liver could be assessed by the level of related hormone in the blood serum. Damage or hepatocyte death could elevate the aspartate transaminase (AST), and alanine transaminase (ALT) and hepatobiliary disorders could interfere with the gamma-glutamyl transferase (GGT) and alkaline phosphatase (ALP) level (13). Also, hematoxylin and eosin (H&E) staining could reveal the histopathology and ultrastructure of the liver. The levels of oxidative and nitrosative stress could be evaluated by different methods in the liver tissue homogenate (14). So, the aim of this study was to evaluate of the bioeffects of exposure to "900/1800 MHz" and “2.4 GHz" RF-EMFs, and x-rays as well as their potential for inducing simple additive, adaptive, or synergistic effects in the liver tissue to contribute to our knowledge about the effects of different types and frequencies of radiation in tissues. Given this consideration, we assessed the oxidative and nitrosative stress markers, histopathology, and ultrastructure and function of the liver. Moreover, the therapeutic effects of intraperitoneal injection of vitamin E were evaluated.

## Methods

**Study design and ethical approval:** This experimental case-control study was done on 120 male Wistar rats, which were randomly divided into ten groups of 12 each. All of the rats were adults and were of the same age, and their weight was in the range of 150 to 200 g. All of the rats were housed in four per polycarbonate cage, and the temperature (25º c) and light-dark cycle (7:00 to 19:00) were the same for all of them. Food and water were always available to rats, and all experiments were performed from 9 to 12 noon. All of the procedures were in the concordance of international guidelines of the care of animals and the study approved by the Ethic Committee of Babol University of Medical Sciences, Babol, Iran. The rats were either exposed to sham, mobile phone, 0.1 Gy, both of 0.1 Gy and GSM 900/1800 MHz RF-EMF and 2.4GHz RF-EMF then they were exposed by lethal dose 50% (LD50/30) or vitamin E + 8 Gy. 100 mg/kg/day (15) vitamin E (injectable solution, dose 100 IU\1 ml, OSVE, Iran) was injected intraperitoneally. The exact grouping was as follows and the treatment procedure was explained in the following sections; 1) Sham control 2) “Mobile phone control” 3) “0.1 Gy control” 4) 8 Gy 5) “Mobile phone + 8 Gy” 6) “0.1 + 8 Gy” 7) “Mobile phone + 0.1 Gy + 8 Gy” 8) “vitamin E + 8 Gy” 9) “Wi-Fi control” 10) “Wi-Fi + 8 Gy”

**Irradiations: **The GSM 900/1800 MHz RF-EMF irradiations were done following our previous studies (16). Briefly, the rats were exposed to a Nokia (model 1280, Nokia India) in the center and three cages on the radius of a circle. Then the phone was in the talk mode for 12 hours a day for 14 days, received the 8 Gy challenging dose on day 15 of the study. The estimated specific absorption rate (SAR) in this situation was 5.57 mW/kg. The 2.4 GHz RF-EMF was exposed following our previous studies (16). Briefly, the rats were exposed by a commercial router (D-Link DSL-2740U ADSL2 Plus Wireless N300 Modem Router, China) in the center and three cages on the radius of a circle. Then the router was on for 12 hours a day during 14 days then, received the challenging dose. The estimated SAR in this situation was SAR 91.99 mW/kg.The exposure of rats to x-ray was done using 6MV Elekta compact accelerator. 10cGy x-ray, as an adaptive dose, was applied at the beginning of the study. The technique of 10 cGy exposure was as the following: the dose rate was 200 cGy/min, SSD=105.5 cm, Depth=3.5cm, MU=11, and field size was 40×40cm. The challenging dose was delivered on day 15 of the study. Delivery condition of eight Gy challenging dose as the LD50/30 of the rats was as follows: the dose rate was 200 cGy/min, SSD=115cm, Depth=3.5cm, MU=983, and field size was 40×40cm. 

**Biochemical evaluations:** Two to three ml of blood was collected from the rats within 24 hours after receiving the 8 Gy ionizing radiation, and after centrifugation, their serum was stored at -80ºC until evaluation. Then the samples were evaluated for GGT, AST, ALT, and ALP by Cobas Mira analyzer.

**Oxidative stress markers:** The rats were anesthetized using ketamine, and they were sacrificed by cervical dislocation. Then, a small part of their liver was excised, weighed, and stored in foiled packets in liquid nitrogen. The other part was stored for histopathological study. Then, based on previously explained protocol (17), tissue homogenates of the first part was obtained by 1/10 (w/v) with phosphate-buffered saline (10 mmol/L, pH 7.4) using a homogenizer (IKA Werke Ultra‐Turrax T25 basic homogenizer; IKA Werke, Breisgau, Germany). Then, centrifugation was done at 10000g for 10 minutes. The samples were stored at -20ºC untile evaluation. Then, the malondialdehyde (MDA), nitric oxide (NO), ferric reducing antioxidant power (FRAP), total thiols (TSH), and protein carbonyl (PCO). We have measured the total protein content of each sample by the biuret reaction. 15µl of samples were added to 600µl of the solution, and after shaking and 10 minutes placement at room temperature, the content was determined in 540nm. This index was used to present the PCO in the mg protein unit. The levels of MDA in the liver tissue were measured as described elsewhere. Briefly, 250 µl of the sample was added to 1250 µl of the MDA solution. Then, after placement in boiling water for 30 minutes, the samples were centrifuged for 5 minutes at 10000g. Then, using the standard curve, which was linear between 0 to 0.35 μmol/mg tissues, the reaction of thiobarbituric acid (TBA), was measured. 

The levels of nitrite were measured by the Griess reaction (18). Briefly, 100µl of samples were added to 100µl of Griess solution, and after 30 minutes placement in 37ºc, the readout was plotted in a standard curve which was linear between 0 to 100μmol/L sodium nitrite and expressed as μmol/mg tissue. The FRAP of samples was done by the colorimetric method (18). 33µl of samples were added to 1 ml of FRAP solution and vortexed. Then, after 10 minutes of incubation, the absorption was read at 590 nm. The content was expressed as μmol/mg tissue using a standard solution.

The TSH was processed by adding 25µl of samples to 150µl of Tris-EDTA buffer and 790 µL of absolute methanol and 10 µL of 10 mM 5,5′‐dithiols‐(2‐nitrobenzoic acid) (DTNB). After mixing gently, the samples were incubated for 15 minutes in room temperature followed by 10 minutes centrifugation in 3000g, the absorbance was read at 412nm against DTNB blank and blank tube, and the concentration was calculated using the molar absorption coefficient of 13600 M^-1^cm^-1 ^(19). The samples were treated with 2, 4-dinitrophenylhydrazine (DNPH), and incubated for 60 minutes in the dark while the tubes were shaken every 15 minutes to measurements of PCO. Then, 200µl trichloroacetic acid (TCA) was added, and after centrifugation, 500µl ethanol: ethyl acetate (1:1) was added, and the guanidine hydrochloride was added to sediment and placed in room temperature for 15 minutes. Then, using the molar absorption coefficient of 22000M^-1^cm^-1^, the PCO was calculated in µmol/mg protein (17).

**Pathological evaluations:** When rats were sacrificed, the right lobe of liver excised and fixed in the buffered formalin 10 %. Then, the samples were dehydrated and embedded in paraffin and stained with hematoxylin and eosin (H&E) (20). Then, the sections were evaluated for possible radiation-induced damages by a light microscope in different magnifications. The observed damages were listed as fibrosis, inflammation, hemorrhage, microvesicular steatosis, macrovesicular steatosis, lobular inflammation, apoptosis, necrosis, sinusoidal dilatation, ductular reaction, and portal or central vein dilation. The evaluation process was blinded by coding the samples separately.

**Statistical analysis:** The mean and standard deviation (SD) of biochemical and oxidative stress parameters were compared by the Kruskal-Wallis test by SPSS software v. 12. Also, Dunn's multiple comparisons tests were done to evaluate the multiple means of different groups. To analyze the results of the histopathological assay of the liver, we used the chi-square test, and the p values lesser than 0.05 were considered significant. 

## Results

**Figure 1:**
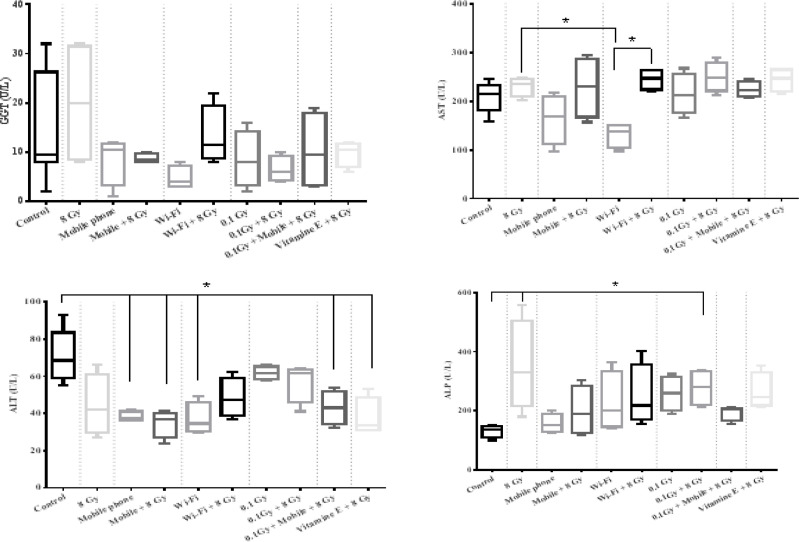
the assessment of GGT (top- left), AST (top- right), ALT (below- left), and ALP (below- right) in the blood serum. The dotted lines represent the different intervention, which was compared with each other and with the control and 8 Gy groups. The comparison of mobile phone+8 Gy, Wi-Fi+8 Gy, 0.1Gy+8Gy, and 0.1Gy+mobile phone+8Gy was not significantly different from each other for all four parameters

**Biochemical evaluations:** The biochemical evaluation of the levels of liver function tests, including GGT, AST, ALT, and ALP in the blood serum after receiving the RF-EMF, x-ray, and their combination, were shown in figure 1. The effects of 8 Gy challenging dose were compared separately after exposure to adaptive doses of GSM 900/1800 MHz RF-EMF, x-ray, 2.4GHz RF-EMF as follows:

1) GSM 900/1800 MHz RF-EMF effects: The changes in the levels of GGT, AST, ALT, and ALP were compared among the controls, 8 Gy, mobile phone, and mobile phone+8 Gy groups.

2) 2.4GHz RF-EMF effects: The changes in the liver function parameters among control, 8 Gy, Wi-Fi, and Wi-Fi +8 Gy groups were compared.

3) X-ray effect: The changes in the mentioned parameters among the controls, 8 Gy, 0.1 Gy, and 0.1 Gy +8 Gy groups were analyzed.

4) The additive, synergic, or AR of GSM 900/1800 MHz RF-EMF and x-ray were analyzed by comparing the mobile phone+0.1Gy+8Gy with controls, and 8 Gy groups.

**Figure 2:**
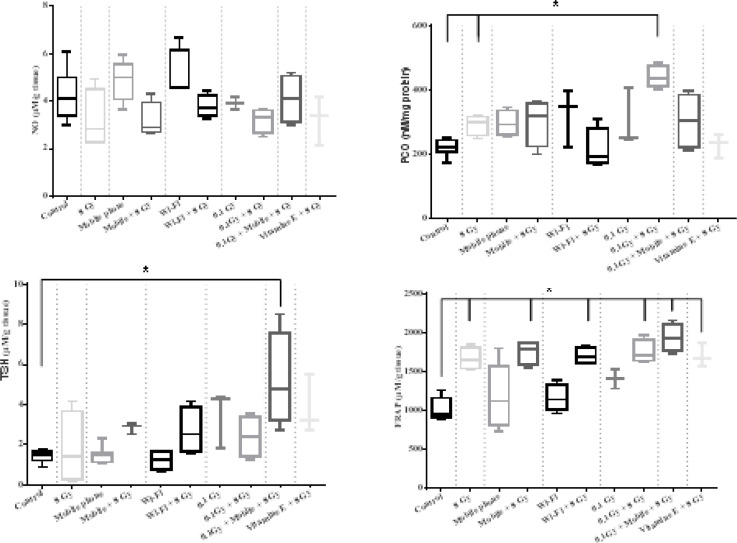
Oxidative stress markers including MDA (top left), NO (top middle), FRAP (top right), TSH (lower left), PCO (lower right) in the liver homogenate

**Oxidative stress markers:** The evaluation of oxidative stress markers including MDA, NO, FRAP, TSH, and PCO in the liver tissue after receiving GSM 900/1800 MHz RF-EMF, x-ray, 2.4GHz RF-EMF, and their combination were shown in figure 2. Pre and post-exposure to adaptive doses of RF-EMFs, and x-ray were compared before and after receiving an 8 Gy challenging dose, separately as follows:

1) GSM 900/1800 MHz RF-EMF effects: The changes in the MDA, NO, FRAP, TSH, and PCO were compared among control, 8 Gy, mobile phone, and mobile phone+8 Gy groups.

2) 2.4 GHz RF-EMFeffect: The changes in the oxidative stress markers among control, 8 Gy, Wi-Fi, and Wi-Fi +8 Gy groups were compared.

3) X-ray effect: The changes in the mentioned markers among control, 8 Gy, 0.1 Gy, and 0.1 Gy +8 Gy groups were analyzed.

4) The possible addition, synergic, or AR of GSM 900/1800 MHz RF-EMF and x-ray were analyzed by comparing the mobile phone+0.1Gy+8Gy with control, and 8 Gy groups.

**Figure 3:**
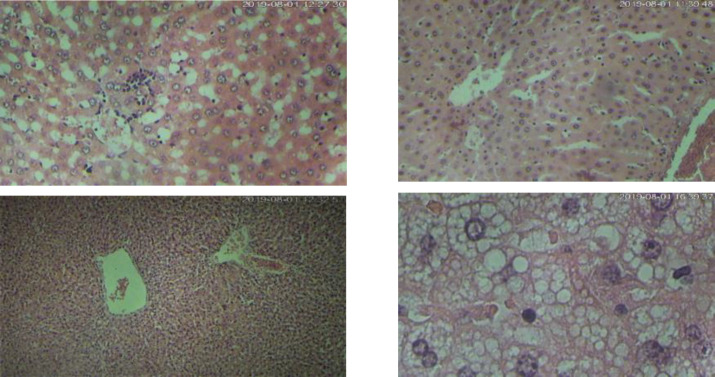
the pathological evaluation of the liver after receiving different agents reviled some alterations. This panel shows a normal (top left), lobular inflammation and apoptosis (top right), central vein dilatation (below left), and micro-vesicular steatosis (below right). Hematoxylin and eosin staining (magnification, x100, and x 400).

**Histopathological** **evaluations:** The histopathological changes of the liver due to different interventions, including GSM 900/1800 MHz RF-EMF, 2.4GHz RF-EMF, x-ray, and their combinations were studied, separately. The illustrations of changes were shown in figure 3. Also, the frequencies and percentages of different histologic changes were shown in table 1.

**Table 1:** the results of histopathological evaluation of the liver after receiving different interventions

	**Central and/or Portal vein Dilatation**		**Portal Inflammation**		**Lobular Inflammation**		**Macro or Micro steatosis**		**Apoptosis or Necrosis**		**Fibrosis**		**Ductular Reaction**		**Hemorrhage**		**sinusoidal Dilatation**	
	No %	Yes %	No %	Yes %	No %	Yes %	No %	Yes %	No %	Yes %	No %	Yes %	No %	Yes %	No %	Yes %	No %	Yes %
Sham Control	5	3	4	4	8	0	7	1	8	0	8	0	8	0	8	0	8	0
	62.5	37.5	50	50	100	0	87.5	12.5	100	0	100	0	100	0	100	0	100	0
8 Gy Control	2	6	5	3	2	6	7	1	5	3	7	1	7	1	7	1	5	3
	25	75	62.5	37.5	25	75	87.5	12.5	62.5	37.5	87.5	12.5	87.5	12.5	87.5	12.5	62.5	37.5
Mobile + 8 Gy	2	6	5	3	5	3	5	3	7	1	7	1	6	2	7	1	6	2
	25	75	62.5	37.5	62.5	37.5	62.5	37.5	87.5	12.5	87.5	12.5	75	25	87.5	12.5	75	25
0.1 + 8 Gy	4	3	6	1	2	6	4	4	5	3	8	0	6	2	7	1	5	3
	57.2	42.8	86	14	25	75	50	50	62.5	37.5	100	0	75	25	87.5	12.5	62.5	37.5
Mobile + 0.1 Gy + 8 Gy	1	7	4	3	2	6	6	2	7	1	7	1	7	1	6	2	3	5
	12.5	87.5	57.2	42.8	25	75	75	25	87.5	12.5	87.5	12.5	87.5	12.5	75	25	37.5	62.5
Vitamin E + 8 Gy	2	6	8	0	3	5	7	1	6	2	8	0	7	1	8	0	5	3
	25	75	100	0	37.5	62.5	87.5	12.5	75	25	100	0	87.5	12.5	100	0	62.5	37.5
Wi-Fi + 8 Gy	1	6	7	0	4	3	2	5	4	3	6	1	5	2	5	2	6	1
	14	86	100	0	57.2	42.8	28.5	71.5	57.2	42.8	86	14	71.5	28.5	71.5	28.5	86	14
Total p value	0.20		0.11		0.02*		0.09		0.34		0.76		0.74		0.54		0.18	
Mobile p value	0.202		0.842		0.008*		0.364		0.122		0.580		0.319		0.580		0.171	
Wi-Fi p value	0.114		0.095		0.009*		0.018*		0.112		0.553		0.261		0.261		0.137	
0.1 Gy p value	0.272		0.343		0.002*		0.135		0.135		0.352		0.319		0.580		0.135	
Mobile+0.1+8Gy p value	0.087		0.880		0.002*		0.741		0.122		0.580		0.580		0.319		0.028	
Vitamin E p value	0.202		0.073		0.006*		1.000		0.171		0.352		0.580		0.352		0.135	

## Discussion

In this case-control study, we have evaluated the impact of RF-EMF 900/1800 MHz, RF-EMF 2.4 GHz, x-ray, and their adaptive or synergistic ability. We have assessed the oxidative and nitrosative stress markers, histopathology, and ultrastructure, and the functional marker of the liver with and without lethal doses of x-ray. Our results are indicating that RF-EMF exposure could alter some properties of the liver.

**Liver function marker:** We have evaluated the function of the liver within 24 hours after 8 Gy irradiation. Our results showed that the GGT levels were not associated with 2.4GHz RF-EMF, GSM 900/1800 MHz RF-EMF, x-ray exposures, and their combinations. Đinđić et al. have assessed the changes in the levels of serum concentration of liver enzymes after receiving 900 MHz RF-EMF, and their results showed that GGT is not affected that is in agreement with our study (21). Then, this marker may not be appropriate to assess radiation-induced liver damage in these doses.

We have observed that the AST levels were not significantly different after receiving 900 MHz and 2.4 GHz radiation. Nevertheless, as we expected, the AST level in the Wi-Fi group was lower than the 8 Gy group because of liver injury caused by this lethal dose. The impact of RF-EMF exposure on the AST level was reported in different studies. Moussa has reported that 3.5 GHz exposure after two weeks did not alter the AST and ALT levels, but the levels were changed after four weeks (22). 

Đinđić et al. (21) showed that the AST level increased after a three-month exposure with 900 MHz irradiation. As the AST and specifically the ALT will be released in response to liver damage and (23) the results of past mentioned researches show that the long term exposure to RF-EMF could be dangerous for the liver. Then, our results indicate that after two weeks exposure of 900MHz and 2.4 GHz RF-EMFs, the excretion of the AST and ALT was not signifying liver damage, even if the levels dropped, which could be a signal to the beneficial effect of this dose on liver function. Nevertheless, after receiving 8 Gy ionizing radiation, ALT did not increase significantly, which could be due to AR. The ALP levels significantly increased after exposure to 8 Gy ionizing radiation. Elevated levels of the ALP, ALT, and AST were reported in different studies due to ionizing radiation (8), which is in agreement with our results. We observed that exposure to GSM 900/1800 MHz and 2.4 GHz RF-EMFs did not change the ALP level, which is similar to Moussa’s (22) study results. Interestingly, our results showed that the ALP was modulated by pre-exposure to 900 MHz and 2.4 GHz RF-EMFs, which is another sign of EMF-induced AR. 

Our results showed that the alteration in the enzyme levels of liver function could be modulated by vitamin E consumption, and pre-exposure to 100-mSv ionizing radiation could be helpful, too. However, we have evaluated the enzyme levels within 24 hours after 8 Gy irradiation. We suggest the serial evaluation in different periods after exposure to confirm our results. If similar results were observed in the long term, we could conclude that some doses of RF-EMF could induce AR in liver function.

**Oxidative stress markers:** We have assessed MDA, NO, FRAP, TSH, and PCO in the liver tissue after receiving 900MHz and 2.4 GHz RF-EMFs and their combinations with ionizing radiation. The effect of vitamin E and pre-exposure to 100-mSv x-ray were evaluated, too. 

The MDA test revealed that the lipid peroxidation increased after receiving 8 Gy ionizing radiation, which was in agreement with other studies (24). Nevertheless, both 900 MHz and 2.4 GHz RF-EMFs did not change the MDA levels at this dose. The higher doses or more prolonged period exposure to RF-EMF caused the MDA to increase in other studies (21), but a shorter period may not alter the MDA in liver tissue (25). Our result showed that pre-exposure to RF-EMF frequencies or a 100-mSv adaptive dose of x-ray could modulate the MDA level after receiving 8 Gy ionizing radiation. However, a combination of 100mSv and RF-EMF before 8 Gy challenging dose, did not represent this modulating effect, which could be due to not being in the adaptive window for this endpoint. The retroperitoneal injection of vitamin E was not effective in radiation-induced lipid peroxidation.

Our result showed that NO levels were not significantly different among the study groups. Some other studies showed that this marker could be associated with RF-EMF exposure. However, our results indicated that short-term exposure to RF-EMF might not interfere with NO level in liver tissue. Besides, FRAP levels the increased after exposure to ionizing radiation, and each pre-treatment did not modulate the effect of challenging dose. The PCO was raised by a single 8 Gy dose in the liver tissue, and similar to the MDA level, it did not increase when the rats were pre-exposed to RF-EMF. This result indicated that the lipid peroxidation and protein oxidation could be involved in AR. The stress aroused by lipid and protein oxidation could cause p53 subcellular localization, which eventually leads to homeostasis (26). Then, the P53 pathway could be involved in AR that was observed in our study. The other pathways with the capability to induce AR in response to oxidative stress are NRF2 and NF-KB (27, 28). Paraswani et al. reported that there is a significant difference between 100mGy and 2Gy exposure in the levels of these proteins, indicating involvement in the AR process (28).

**Histopathological changes:** The fibrosis, inflammation, hemorrhage, microvesicular steatosis, macrovesicular steatosis, lobular inflammation, apoptosis, necrosis, sinusoidal dilatation, ductular reaction, and portal or central vein dilation were analyzed, and their associations with our interventions were hypothesized. The frequency of lobular inflammation was associated with interventions. Our results showed that lobular inflammation in the liver tissue is an outcome of the exposure to lethal doses of ionizing radiation. Julie A. Reisz et al. reported that oxidation is associated with the progression of inflammation (29). Also, oxidative stress could lead to inflammation, apoptosis, and eventually fibrosis in the nonalcoholic fatty liver disease (30). We have shown the PCO and other oxidative stress markers are changed by irradiation. Upregulation of the pathways such as NRF2 could lead to AR in the inflammatory process (31), and the changes in biochemical tests could be due to these alterations. In conclusion,** o**ur results showed that the exposure to both ionizing and non-ionizing radiations could alter some liver function tests. A short term pre-exposure to RF-EMF then irradiation with an 8 Gy challenging dose of x-ray showed that oxidative stress could be involved in the adaptive response. This oxidative stress, induced by RF-EMF, eventually causes the enhanced function of the irradiated liver with ionizing radiation. The literature suggests the involvement of oxidative stress and inflammation-related pathways in this process, but their exact mechanisms need to be examined in detail.

## Funding:

The Deputy for Research and Technology of Babol University of Medical Sciences, Babol, Iran, approved and financially supported this study.
